# Tamoxifen-Loaded Eudragit Nanoparticles: Quality by Design Approach for Optimization of Nanoparticles as Delivery System

**DOI:** 10.3390/pharmaceutics15102373

**Published:** 2023-09-22

**Authors:** Muzna Ali Khattak, Zafar Iqbal, Fazli Nasir, Steven H. Neau, Sumaira Irum Khan, Talaya Hidayatullah, Sadia Pervez, Mirina Sakhi, Syeda Rabqa Zainab, Shazma Gohar, Fawaz Alasmari, Altafur Rahman, Gul e Maryam, Arbab Tahir

**Affiliations:** 1 Department of Pharmacy, University of Peshawar, Peshawar 25120, Pakistan; muznaali@uop.edu.pk (M.A.K.); talayaarbab@uop.edu.pk (T.H.); sadiapervez@uop.edu.pk (S.P.); syedarabqazainab@uop.edu.pk (S.R.Z.); shazmagohar@gmail.com (S.G.); altafrkhanutmanzai@gmail.com (A.R.); gulemaryam112@gmail.com (G.e.M.); tahir.arbab@uop.edu.pk (A.T.); 2 Department of Pharmacy, Cecos University of IT and Emerging Sciences, Peshawar 25000, Pakistan; 3 Department of Pharmacy, Sarhad University of Science and Information Technology, Peshawar 25000, Pakistan; dean.fls@suit.edu.pk; 4 Philadelphia College of Pharmacy, University of Sciences, Philadelphia, PA 19104, USA; stevenneau@yahoo.com; 5 Pharmacy Department, Faculty of Health and Medical Sciences, Mirpur University of Science and Technology, New Mirpur City 10250, Pakistan; sumairairumkhan@gmail.com; 6 Department of Pharmacy, University of Swabi, Swabi 23430, Pakistan; mirina.sakhi@yahoo.com; 7 Department of Pharmacology and Toxicology, College of Pharmacy, King Saud University, Riyadh 11362, Saudi Arabia; ffalasmari@ksu.edu.sa

**Keywords:** drug delivery, Eudragit RS 100, tamoxifen, design of experiment, response surface methodology, statistical analysis, nanoparticles

## Abstract

Nanoparticles have numerous applications as drug carriers in drug delivery. The aim of the study was to produce tamoxifen nanoparticles with a defined size and higher encapsulation for efficient tissue uptake with controlled drug release. The quality by design approach was utilized to produce tamoxifen-loaded Eudragit nanoparticles by identifying the significant process variables using the nanoprecipitation method. The process variables (amount of drug, polymer, and surfactant) were altered to analyze the influence on particle size (PS), % encapsulation efficiency (EE). The results showed that the drug and polymer individually as well as collectively have an impact on PS, while the surfactant has no impact on the PS. The %EE was influenced by the surfactant individually and in interaction with the drug. The linear regression model was endorsed to fit the data showing high R^2^ values (PS, 0.9146, %EE, 0.9070) and low *p* values (PS, 0.0004, EE, 0.0005). The PS and EE were confirmed to be 178 nm and 90%, respectively. The nanoparticles were of spherical shape, as confirmed by SEM and TEM. The FTIR confirmed the absence of any incompatibility among the ingredients. The TGA confirmed that the NPs were thermally stable. The in vitro release predicted that the drug release followed Higuchi model.

## 1. Introduction

For multimodal treatment, the nanotechnology-based treatment approaches showed promising results, due to their high potential to improve the delivery of chemotherapeutic agents to cancerous cells while minimizing its distribution to normal cells [1,2,3,4,5]. The limitations related to chemotherapy can be overcome by employing the strategies of the nanomedicine to the anticancer drug formulations by using several nanocarriers [6,7,8,9]. For nano and microcarriers, polymers are the most reliable materials [10]. Apart from the route of administration, particle size, shape and surface charge and functional group play a key role in the controlled uptake of polymeric nanoparticles [11]. It is essential to control the modification of particle properties, depending on the target cell, to deliver the drug to the desired target site. For the development of these tailored particles, it is crucial to understand the process of preparation and the variables that influence the final product. This allows the management and control of production [12].

The production method still faces multiple challenges, such as control and replication of the required nanoparticles, which make it difficult to generate high-quality PNP (polymeric nanoparticles) despite major significant advances in laboratory-scale PNP preparation. PNP preparation procedures are frequently multi-step bulk processes with numerous affecting variables, making the preparation quite difficult. The use of quality by design (QbD) is crucial and requested by ICH to guarantee the final quality of the product early in the development of the technique and to understand as early as is feasible what factors influence the process. Within this frame of reference, designing Eudragit NPs of TAM that have a small particle size and high encapsulation efficiency optimized by factorial design is notable [13].

Quality by design is an analytical approach used in the development of pharmaceuticals. It entails evaluating and understanding the manufacturing and formulation processes. The objective is to develop a controlled procedure that ensures the product’s quality. Different methods and tools to implement QbD in practice are described in the ICH guideline Q8(R2), including “multivariate experiments”, “statistical process control methods”, also known as design of experiments, and a “risk-based control strategy [14]”.

The present study shows the application of the QbD by development of tamoxifen loaded Eudragit nanoparticles. The Eudragit RL is cationic copolymer derived from acrylic acid and methacrylic acid esters with quaternary ammonium groups [15]. Nowadays, the Eudragit RL and RS polymers are the pharmaceutical’s industry preferred choice for sustained drug release profiles due to their pH-independent swelling characteristics [16,17]. Various factors including the concentration of the drug, polymer, surfactant and solvent highly influence the particle size and %EE. When these factors are controlled, the nanoparticle of the desired profile can be prepared [18].

Tamoxifen is a non-steroidal antiestrogen and a selective estrogen receptor modulator. It has been clinically used for more than 20 years for the antiestrogenic therapy of malignancy or advanced breast cancer [19]. It has been used as an additional therapy following post-menopausal cancer and primary treatment of early-stage breast cancer [20]. Tamoxifen showed 20–30% oral bioavailability due to an extensive intestinal and hepatic first-pass effect, so the requirement of the dose is increased along with chronic (long-term) duration of therapy [21,22,23,24,25]. Tamoxifen is associated with multifocal hepatic fatty infiltration, toxic hepatitis and hepatic necrosis and cirrhosis [26]. Tamoxifen is also associated with increased risk of endometrial cancer, which is mainly due to its long-term treatment and dose accumulation [24]. Therefore, an alternate therapy is required for optimal chronic administration of tamoxifen with enhanced bioavailability and reduced adverse effects (hepatotoxicity).

This paper presents the utilization of QbD analysis and optimization for the development of tamoxifen-loaded Eudragit nanoparticles. In this study, the effects of multiple factors are being studied. The factorial experimental design is ideal for this type of study. The mathematical model included in this study evaluates the linear effects of the multiple factors as well as the effects of their interactions [27]. Compared with OFAT, factorial design provides more information and finds optimal conditions faster than OFAT experiments. OFAT experimental design cannot detect if the effect of one factor is different for different levels of another factor. To detect such interactions, factorial design is obligatory [28,29]. Identifying the process variables that have an impact on the finished product will be enough to change its attributes in a targeted manner, which is crucial for guided optimization. The study was divided into five phases.

- The planning phase includes the selection of responses (outcome parameters) and identifying and assessing process variables that can affect the characteristics of Eudragit-NP.
- The screening phase includes the screening of the most promising variables to identify relevant process parameters and the first strategy for optimization using the screening findings.
- The optimization phase includes controlled modification of final product quality by altering the most impactful parameters within the RSM (response surface methodology)
- The verification phase includes prediction and confirmation of the ideal process variables.

## 2. Materials and Methods

### 2.1. Chemicals

Tamoxifen citrate (purity 99.9%), purchased from Huzhou Zhanwang Pharma Co., Ltd., Huzhou, China, Eudragit RS 100 (Evonik, Essen, Germany); Acetonitrile (purity > 99.9%) was purchased from Fisher Scientific PVT. U.K. Ltd. (Loughborough, UK), Methanol (purity > 99.9%), Sodium dodecyl sulphate, Dialysis Tubing-Visking, (MWCO; 12–14 kDa); (Dia = 27/32″–21.5 mm); (Size 6 Inf. 30 M) (Sigma-Aldrich), distilled water [Millipore ultrapure water sys (Milford, CT, USA)].

### 2.2. Design of Experiments

A screening study was performed to screen the variables to be included in the study prior to the experimental design. The chosen factors of interest determined during the screening studies were varied on two levels along with three center-points (−1, 0, +1) in agreement with the experimental design. The skeptical curvature effects in the current design space were also assessed by including the center points [30,31]. Many significant second-order effects do not influence how main effects are estimated. In addition, if the genuine effects are significantly bigger than the error standard deviation, two-factor interactions or quadratic effects can be approximated [11]. The studied independent factors or inputs were the amount of the tamoxifen, Eudragit, stabilizer. Screening of suitable and optimal stabilizer is a prerequisite to prevent aggregation and stabilization the nanosuspension. A range of stabilizers were varied when preparing nanoparticles and different nano formulation were prepared by varying the type of stabilizer at constant stabilizer concentration. SDS (sodium dodecyl sulphate) was chosen over Pluronic F127 PVA, SLS, tween 80, PVP (K 30), as a stabilizer based on reduced particle size and high %EE in screening studies. The observed responses or outputs were PS and drug % EE of nanoparticles. A total of 11 experimental runs was suggested using Design Expert v.13.0 software (Stat-Ease, Minneapolis, MN, USA) for optimization of tamoxifen-loaded Eudragit nanoparticles. The coded and actual experimental levels based on a two-level, three-factor approach is given in Table 1, and the design of experiments using Design Expert software is presented in Table 2.

### 2.3. Nanoparticle Preparation

Tamoxifen-loaded polymeric nanoparticles were prepared according to the nanoprecipitation method [32]. Briefly, the drug (TAM) was dissolved in 2 mL methanol, and polymer (Eudragit RS 100) solution was prepared by dissolving it in 5 mL of acetonitrile. Both the solutions were mixed (organic phase) and added dropwise (1.75 mL/min) to 10 mL of stabilizer (sodium dodecyl sulphate) solution (aqueous phase) using a peristaltic pump with continuous stirring. The organic phase was vaporized via overnight stirring.

### 2.4. Purification of Nanoparticles

The nanoparticles were subjected to three centrifugation steps to remove any excess of stabilizer and nonencapsulated drug and to avoid agglomeration. The nanosuspension was centrifuged for 30 min at 10,000 rpm at 4 °C thrice. The clear supernatant was decanted and the nanoparticles containing pellets were resuspended in distilled water and freeze-dried using sucrose as cryoprotectant.

### 2.5. Particle Size and Poly-Dispersibility Index (PDI)

The prepared tamoxifen-loaded nanoparticles were evaluated using a dynamic light scattering technique for PS and PDI using a Zeta-Sizer (Nano ZS-90, Malvern Instru., Malvern, UK) with an equilibration time of 120 s at 25 °C. All the readings were taken in triplicates and the mean ± SD was calculated.

### 2.6. Zeta Potential

The zeta potential of the reconstituted nanoparticles was measured using a Zeta-Sizer (Nano ZS-90, Malvern Instruments, Malvern, UK) by Laser Doppler Micro- Electrophoresis. Mean ± standard deviation (SD) was calculated by taking measurements in triplicate.

### 2.7. Encapsulation Efficiency and Drug Loading

The % encapsulation efficiency (EE) of tamoxifen was determined by centrifuging the nanoparticles at 5000 rpm at 4 ℃ for 30 min (Centurion^®^ Scientific, Chichester, UK). The amount of free drug was determined in the supernatant according to the developed HPLC method. The following formula was used to calculate the % encapsulation efficiency and drug loading [33].

$$\%\mathrm{EE}=\frac{amount\;of\;drug\;entraped}{total\;amount\;of\;drug\;used}\times 100$$

$$\%\mathrm{DL}=\frac{weight\;of\;the\;drug\;in\;nanoparticles}{weight\;of\;the\;nanoparticles}\times 100$$

### 2.8. Scanning Electron Microscope (SEM)

SEM (JSM-5910, JEOL, Tokyo, Japan) analysis was utilized for the characterization of surface morphology and shape of drug-loaded nanoparticles. For the SEM analysis, the lyophilized nanoparticles were spread on the adhesive carbon tape attached to the stub. The nanoparticles surface was coated with gold (Au) via a coater (Argon Sputtering, SPI Module Control) for about 90 s under a vacuum. The prepared sample was then analyzed under SEM.

### 2.9. Transmission Electron Microscope (TEM)

TEM analysis was utilized to obtain the crystallographic, morphologic, and compositional information of drug-loaded nanoparticles. The sample was placed on the carbon grid in the form of nanosuspension and the image was obtained.

### 2.10. X-ray Diffraction (XRD)

The XRD pattern of the pure drug, polymer, stabilizer, and their physical mixture in 1:1 and optimized formulation was studied at an angular range (2θ) of 10°–40° using a JDX-3532 X-ray diffractometer (JEOL, Japan).

### 2.11. Thermo-Gravimetric (TGA) Analysis

TGA analysis was performed to assess the phase transition, thermal decomposition, and solid gas reactions (oxidation, reduction). The experiment mass was monitored throughout the experiment. A sample purge gas (inert, reactive) controlled the sample environment.

### 2.12. In Vitro Drug Release

The optimized nanoformulation was assessed to determine its drug-release profile as per ICH guidelines, the samples were analyzed in triplicate. A Franz cell utilizing a dialysis membrane (Dialysis Tubing-Visking MWCO: 12–14 kDa) was used to determine the in vitro release profile of tamoxifen from polymeric nanoparticles. An appropriately conditioned dialysis membrane was mounted on the Franz diffusion cell. The acceptor compartment was filled with the dissolution media (phosphate buffer pH 7.4) and stirred at 600 rpm throughout the study. The acceptor compartment was covered with a jacketed vessel consisting of an inlet and an outlet to which a peristaltic pump was attached for circulation of hot water to maintain the temperature at 37 °C. The calculated amount of nanosuspension was placed on the donor compartment and closed using parafilm to prevent water evaporation. An aliquot of 0.5 mL was withdrawn at predetermined time intervals (15 min, 30 min, 1 h, 2 h, 3 h, 6 h, 12 h, 24 h, 48 h, 72 h, 96 h, and 120 h) and replaced with equal volume of freshly prepared dissolution medium to sustain the sink conditions. The drug released at various time intervals was assayed by the developed HPLC method.

### 2.13. Drug-Release Kinetics

Various mathematical models were fitted to the accumulative drug release data obtained from the dissolution testing; the mathematical models fitted to data include a zero-order model, first-order model, Hixson and Crowell model, Higuchi model and Korsmeyer–Peppas model.

### 2.14. Stability Studies

Stabilities studies were performed at two different temperatures, i.e., at refrigerated temperature (4 °C) and room temperature (25 °C) to evaluate the storage effect on the PS, PDI, and EE of the nanoformulations.

## 3. Results

It is necessary to specify a target product profile before beginning a QbD experimental setup. Specific requirements are established based on the NP drug carrier systems’ intended use. These requirements are crucial to the selection of variables and later process parameter optimization.

### 3.1. Target Product Profile Set Up

It is crucial that the nanoparticulate carrier delivers enough API (active pharmaceutical ingredient) to or into the target cell and releases it there to produce a therapeutic effect. As was previously stated, when it comes to distribution in the body, PS and %EE are the key quality factors with regard to target cell contact and cellular uptake. For the outcome variable, the z-average (average particle size) was chosen as one of the DOE’s (Design of Experiments) outcome metrics due to its significance in reaching the target cell, along with maximum %EE for process cost effectiveness. A careful analysis of the factors should be conducted to ascertain the variables as completely as possible. The evaluation should focus on a variable’s potential impact on the results. Additionally, to rule out merely theoretical solutions, practicability, effort, and detectability should be considered. To exclusively allow uptake in phagocytosing cells, the ideal NP was defined for this investigation to have a z-average up to 200 nm and % encapsulation efficiency of 90%.

### 3.2. Design of Experiment

A two-level, three-factor full factorial design (2^3^) was used to optimize tamoxifen-loaded polymeric nanoparticles according to the nanoprecipitation method. A total of 11 experiments were carried out as suggested by the Design Expert^®^ software. The procedures were executed in triplicates and the data obtained are shown in Table 3. The statistical significance was judged at α = 0.05 (a *p*-value less than 0.05 indicates statistical significance of the term in or a property of the model equation). The optimized tamoxifen-loaded nanoparticles were then subjected to physiochemical evaluation and in vitro drug release kinetics. The nanoparticles were characterized by their PS, PDI, morphology, EE, stability, and in vitro drug release profile.

### 3.3. Effect on Particle Size

The data obtained as a result of the experiments demonstrated that the concentration of the factor A (Drug) and factor B (polymer) individually as well as in interaction has a high effect on particle size, which was confirmed by statistical analysis of the drugloaded Eudragit nanoparticles.

### 3.4. Statistical Analysis of Particle Size

**Figure 1:**
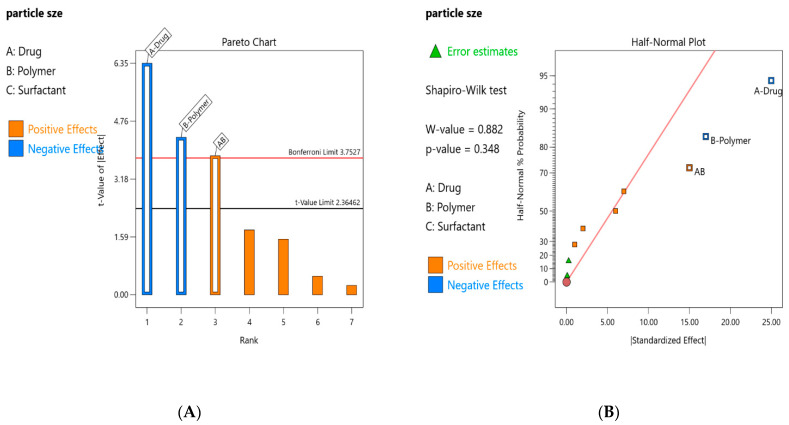
(**A**) (Model analysis) Pareto chart of PS for drug-loaded Eudragit nanoparticles. (**B**) Half-normal plot of PS for drug-loaded Eudragit nanoparticles.

The data obtained as a result of experiments were analyzed via a half-normal plot and a Pareto chart to determine the factors that have profound effects on the particle size. In the half-normal plot, the high effects were identified by their appearance on the right of the line that best fits the collinear data at the lower left of the plot (Figure 1B). The Pareto chart (Figure 1A) was analyzed for the high t- value of the dominant effects and % contribution of the selected factors. The results showed that the % contribution of drug and polymer individually and in their interaction (AB) was 50.18, 23.30, and 18.06, respectively (Table 4). It is depicted from the results that the concentration of the drug has the highest contribution to varying particle size.

### 3.5. ANOVA of Particle Size

The ANOVA (Table 5) indicated that the Model F-value of 24.99 implies the model is significant. The low *p*-value of (0.0004) shows that the linear regression model was highly significant for the experimental data. The lack of curvature and the Lack of Fit F-value of 13.78 implies the fitness of the model. The reliable results of the descriptive statistics (Table 6) show the adequacy of the model. The goodness of fit was depicted by the excellent R2 value of 0.9146, suggesting that the model equation describes the responses reasonably well. The predicted R^2^ of 0.7240 was in reasonable agreement with the adjusted R^2^ of 0.8780; i.e., the difference was less than 0.2. The signal-to-noise ratio of 12.63 (greater than 4) indicates an adequate signal. The low co-efficient of variance value, i.e., 3.61, indicated the high degree of precision and reliability of performed experiments.

### 3.6. Final Equation in Terms of Coded and Actual Factors

By evaluating the results, the linear regression model demonstrated better descriptive statistics with respect to a higher R2 value and low *p*-value. Therefore, the linear regression model was endorsed to fit the experimental data and set up a final model equation to show the correlation between the particle size and formulation variables. The final model equation in terms of the coded and actual term is given in Equations (1) and (2).

PS = +152.55 − 12.50 A − 8.50 B + 7.50 AB(1)

PS = +224.54545 − 27.50000 Drug − 9.40000 Polymer + 3.00000 Drug × Polymer(2)

The model equation indicates that the two factors and their interaction are significant in terms of their influence on particle size; namely, the amount of drug and the polymer.

### 3.7. Model Diagnostic Plot

#### 3.7.1. Normal Plot of Residuals

**Figure 2:**
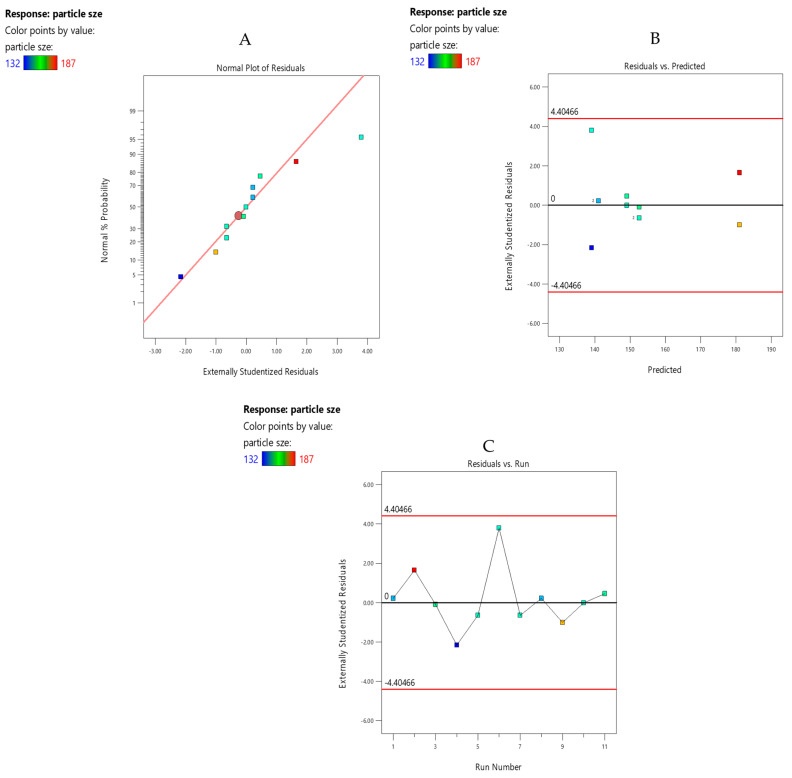
(**A**) Normal plot of residuals of PS for drug-loaded Eudragit nanoparticles. (**B**) Residuals vs. predicted plot of PS for drug-loaded Eudragit nanoparticles. (**C**) Residuals vs. run plot of PS for drug-loaded Eudragit nanoparticles.

The normal plot of residuals (Figure 2A) was analyzed for the magnitude of the effects of each of the main factors and each of the two-factor interactions. The factors that have a profound effect on the particle size were selected. The normal plot of residuals illustrated normal distribution of the data points being close to the linear relation presented on the plot. Hence, the plot was unremarkable.

#### 3.7.2. Residuals vs. Predicted Plots

The relationship between the predicted and experimental value was analyzed by generating predicted and actual plots. There was no outlier and unacceptable pattern found in the plot (Figure 2B). Hence, the plot was unremarkable as it showed the normal pattern of the data points. The residual vs. run plot (Figure 2C) showed a satisfactory goodness of fit, as all the data points were well distributed within the control limits, showing all the experiments were conducted in a random manner confirming adequate fit.

#### 3.7.3. Model Graphs

**Figure 3:**
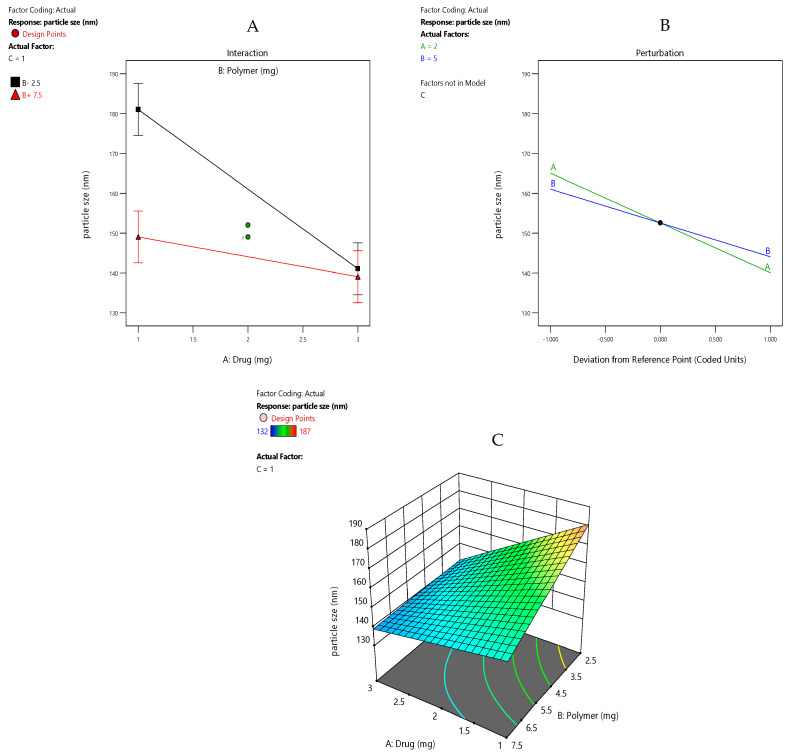
(**A**) Interaction plot of PS for drug-loaded Eudragit nanoparticles. (**B**) Perturbation plot of PS for drug-loaded Eudragit nanoparticles. (**C**) Response surface plot of PS for drug-loaded Eudragit nanoparticles.

The perturbation plot, interaction plot, and 3D response surface plot were generated for the visualization of the main factors and interaction effects that lead to variance in the particle size. The perturbation plot (Figure 3B) presents that the high level of factor A and B decreases the particle size considerably and vice versa, as is evident based on the steep slope of both the factors. The results agreed with the previous literature [34,35,36,37,38,39]. The interactive plot (Figure 3A) displays the interaction between factor A (Drug) and B (polymer). There was no significant difference between the drug concentration at 1 mg (low level) and 3 mg (high level) when the polymer was operated at a high concentration, i.e., 7.5 mg. The high drug concentration that operated at low polymer concentration had a significant effect on the response, i.e., decreased the particle size considerably. The overlapping LSD bars show that the two means are statistically the same between drug concentrations of 1 mg and 3 mg. The 3D response surface plot (Figure 3C) generated for particle size confirmed the same results in the design space.

### 3.8. Effect on %EE

The results of % encapsulation efficiency of Eudragit nanoparticles revealed that the concentration of surfactant individually, as well as in interaction with the drug, has a huge effect on the % EE, as indicated by the low *p*-value of 0.0004 and 0.0013, respectively (Table 8).

### 3.9. Statistical Analysis of %EE

**Figure 4:**
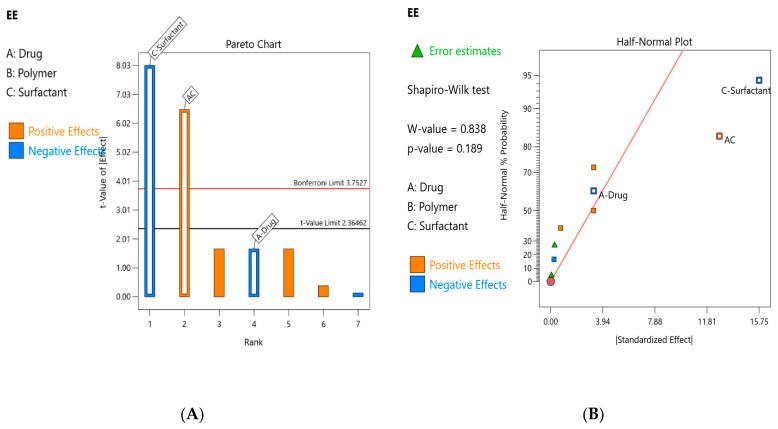
(**A**). Half-normal plot of %EE for drug-loaded Eudragit nanoparticles. (**B**). Pareto chart of %EE for drug-loaded Eudragit nanoparticles.

The size of the effects of each of the core factors and each of the two-factor combinations was assessed using a half-normal plot and a Pareto chart (Figure 4A,B). A numeric chart of the % contribution (Table 7) showed that the % contributions of factor A, C individually as well as that of their interaction are 2.274%, 53.41% and 35.00%, respectively. It was apparent from the results that factor C contributed significantly to the variance in the encapsulation of the drug.

### 3.10. ANOVA of %EE

The ANOVA (Table 8) indicated that the curvature was insignificant. The suggested model (linear regression model) was highly significant with a Model F-value of 22.76 and a *p*-value of 0.0005. The **Lack of Fit F-value** of 16.74 depicts that the model was fit with no curvature. The descriptive statistics (Table 9) confirm the reliability of the model. A high **R-Squared** value (R^2^ 0.9070) indicated that the model equation describes the factorial experiment results well. The **Adjusted R-squared** value (0.8672) was close to that of the R-squared because there were enough degrees of freedom to describe the data well. The **Predicted R-squared** (0.7529) was in reasonable agreement with the adjusted R-squared. The value of adequate precision (13.456) showed that signal-to-noise ratio was good. The low value of coefficient of variance (4.53) showed a high degree of precision and reliability for the conducted experiment.

### 3.11. Final Equation in Terms of Coded and Actual Factors

The high R^2^ value and low *p*-value confirmed that the linear regression model was the best fit for experimental data. The final model equation obtained in the coded and actual variables is given Equations (3) and (4).

EE% = +77.45 − 1.63 A − 7.87 C + 6.38 AC(3)

EE% = +121.95455 − 14.37500 Drug − 206.25000 SDS + 63.75000 Drug × SDS(4)

The model equation indicated that the two factors and their interactions are significant in terms of their influence on %EE; namely, the amount of the drug and surfactant.

### 3.12. Model Diagnostic Plot

#### 3.12.1. Normal Plot of Residuals

**Figure 5:**
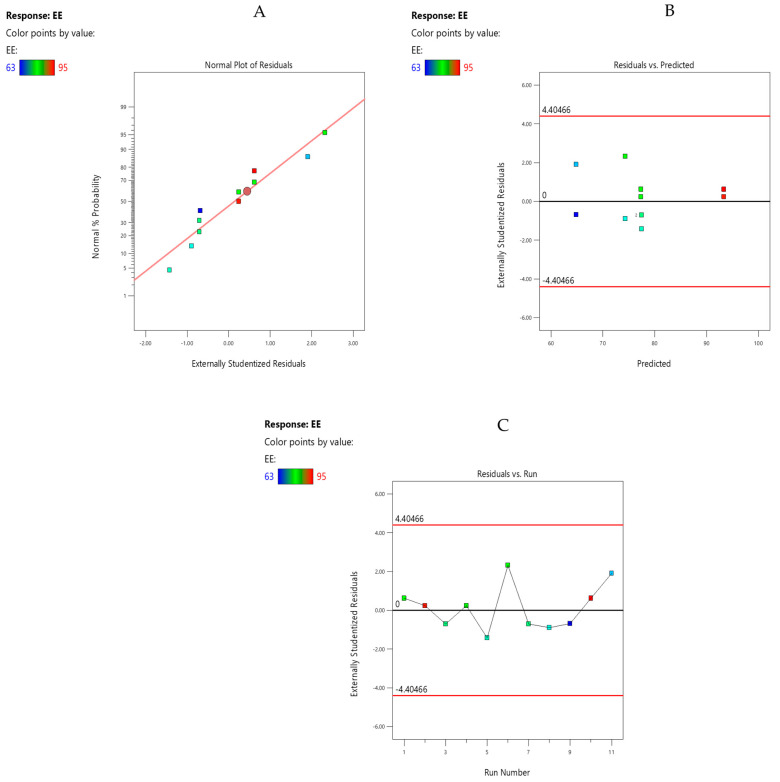
(**A**) Normal plot of residuals of %EE for drug-loaded Eudragit nanoparticles. (**B**) Residual vs. predicted plot of %EE for drug-loaded Eudragit nanoparticles. (**C**) Residuals vs. run plot of %EE for drug-loaded Eudragit nanoparticles.

The plot (Figure 5A) showed normal distribution as all the data points fell close to the linear relation presented on the plot. The plot was unremarkable.

#### 3.12.2. Residuals vs. Predicted Plots

The Residual vs. predicted plot of the % EE showed a normal pattern of the actual data. There was no outlier and unacceptable pattern found in the plot (Figure 5B). Hence, the plot was remarkable. The residual vs. run plot (Figure 5C) showed a satisfactory goodness of fit because all the data points were distributed well within the control limits, showing that all the experiments were conducted in a random manner which confirmed adequate fit.

#### 3.12.3. Model Graphs

**Figure 6:**
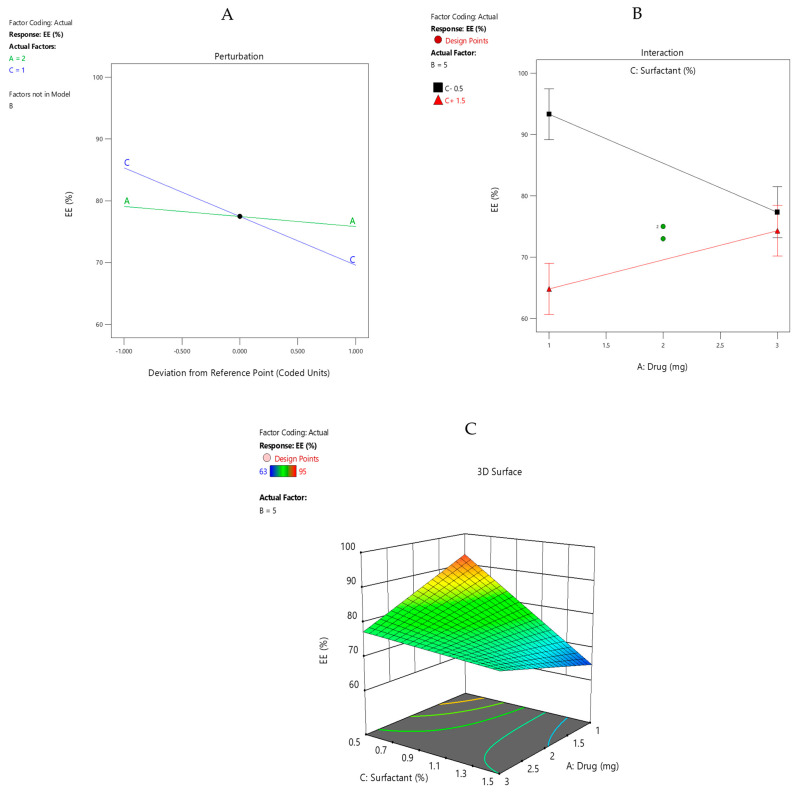
(**A**) Perturbation plot of %EE for drug-loaded Eudragit nanoparticles. (**B**) Interaction plot of %EE for drug-loaded Eudragit nanoparticles. (**C**) Response surface plot of %EE for drug-loaded Eudragit nanoparticles.

The perturbation plot (Figure 6A) of %EE depicted that the drug concentration has no significant effect compared with the surfactant concentration, which affects the %EE considerably. The drug concentrations at low and high concentration overlap with one another, showing no significant effect. At a low surfactant concentration level, the %EE increased considerably and vice versa. The results were in accordance with the previous literature [34]. The interaction plot (Figure 6B) displayed interaction between factor A and C. There was a significant difference between drug concentration at 1 mg and 3 mg when the surfactant was operated at a low concentration. There was a significant increase in % EE when a low drug concentration was operated at a low surfactant concentration and vice versa, which is desirable. The low drug concentration at a high level of surfactant significantly decreased the %EE, which is not desirable. The overlapping LSD bars shows that the two means are statistically the same. The same effects were shown by the response surface plot of %EE (Figure 6C).

### 3.13. Optimization, Validation of the Optimized Condition

The optimization was based on minimizing the particle size with maximum encapsulation efficiency within the range, i.e., from 150 nm to 187 nm and from 63 to 99%, respectively. The developed regression model was used to determine the optimal condition for the preparation of nanoparticles within the given range. An optimized solution for the preparation of the desired nanoparticles was selected among the solutions proposed by the software with the desirability of 0.820. The corresponding formulation parameters for factor A (drug), B (polymer), C (surfactant) were 1 mg, 2.5 mg and 0.1%, respectively (Table 10). The predicted particle size and %EE was 181 nm and 93% under the optimized condition. To carry out the confirmatory runs, the experiment was run in triplicate with optimized conditions to compare the predicted values with the experimental values. Under the optimal condition, the experimental value of the particle size and %EE obtained was 178 nm and 90%, respectively, which matched well with the predicted values. The % DL of the optimized nanoparticles was found to be 43%.

### 3.14. Confirmation of the Results

The confirmation of the results was conducted at 95% confidence. The results are shown in Table 11. The stated objective of the research was achieved by developing tamoxifen-loaded nanoparticles with low levels of all the formulation variables with reduced particle size and high % encapsulation efficiency by factorial design. The concentrations of drug, polymer, and surfactant used in the optimized nano formulation were lower than the reported concentration and under the permissible safety limits [40,41].

### 3.15. Particle Size and PDI

**Figure 7:**
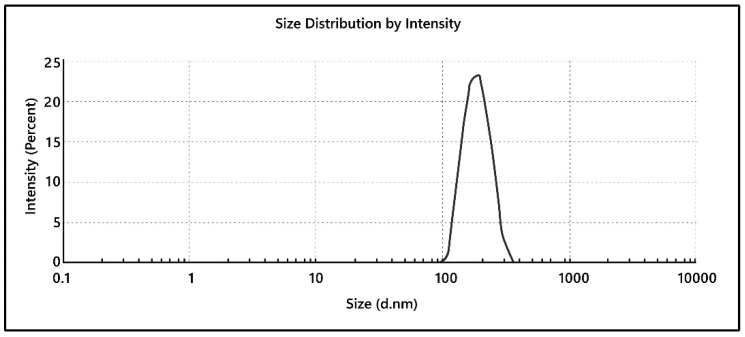
Size and PDI graph of optimized drug-loaded Eudragit nanoparticles.

The particle size and PDI of the optimized Eudragit nanoparticles are shown in Figure 7. The particle size was well matched with the predicted value. The PDI of the optimized formulation was 0.013, confirming the homogeneous and monodisperse nature of nanoformulation. Cells can internalize particles smaller than 100 nm via endocytosis or a clathrin-dependent process. Consequently, these particles provide a greater toxicity risk, and the 100 nm limit must also be taken into account [42,43]. The optimized particle size was 181 nm, which is well within the safety criteria, as demonstrated by earlier studies.

### 3.16. Zeta Potential

**Figure 8:**
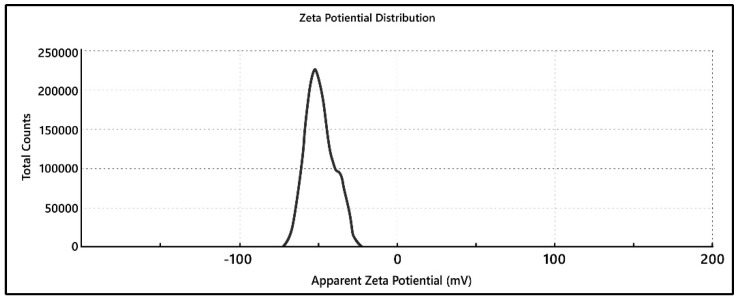
Zeta potential graph of optimized drug-loaded Eudragit formulation.

The zeta potential of the optimized nanoformulation is shown in Figure 8. The zeta potential of the optimized formulation was found to be −48.8, which confirmed that the nanoformulation was highly stable. The high zeta potential indicates that the particles are small enough to resist aggregation due to electrostatic repulsion [27]. Because of the strong electrostatic interaction between charged nanoparticles and cell membranes, positively charged nanocarriers may benefit from the high adhesion with cell membranes, since the cytomembrane is negatively charged due to the surface anionic chemical entities. However, the clathrin-mediated endocytosis route and/or the caveolae-mediated endocytosis route, which are the two most common pathways for cellular internalization of NDDSs, may also be encouraged by the cationic nanocarriers. Additionally, positive surface charge can cause harmful side effects, including tissue/cell toxicity and hemolysis due to the strong interaction with blood serum components and non-specific adhesion with normal cells discussed above [44]. These results are supported by a study conducted by Patil et al. [45]

### 3.17. Scanning Electron Microscopy (SEM)

**Figure 9:**
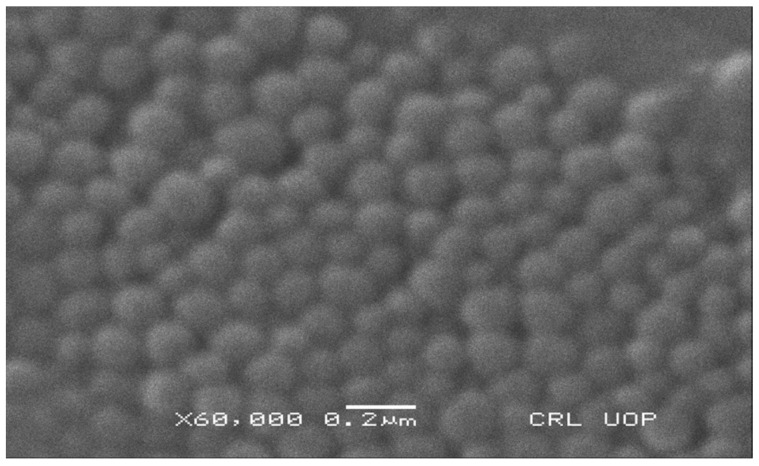
SEM image of drug-loaded Eudragit nanoparticles.

For visual evaluation of the prepared nanoparticles, the SEM image and the surface morphology of the resulting nanoparticles were studied. The SEM image (Figure 9) displayed the uniform spherical shape of the prepared Eudragit nanoparticles with smooth surfaces.

### 3.18. TEM of Drug-Loaded Eudragit Nanoparticles

**Figure 10:**
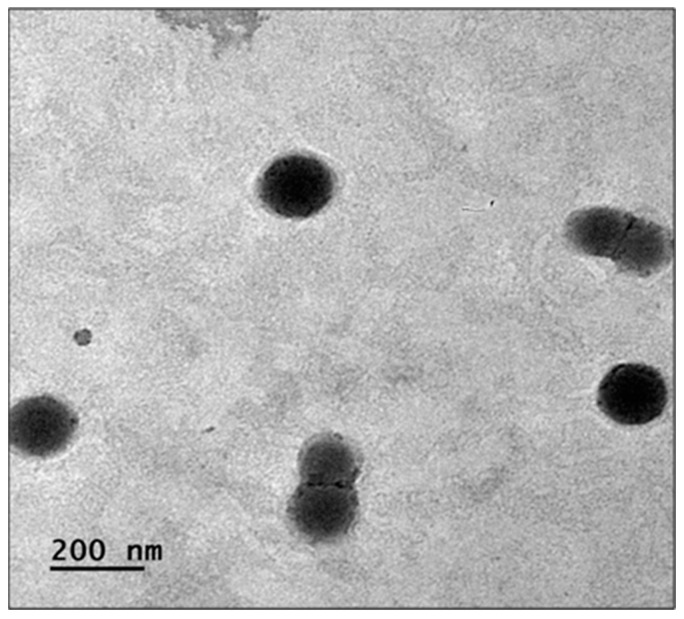
TEM images of drug-loaded Eudragit nanoparticles.

The TEM image (Figure 10) of drug-loaded Eudragit nanoparticles showed aggregates of particles, which were spheroidal (almost rounded) in shape and homogeneous in size. The magnified image of TEM showed the crystalline and porous nature of particles.

### 3.19. X-ray Diffraction

**Figure 11:**
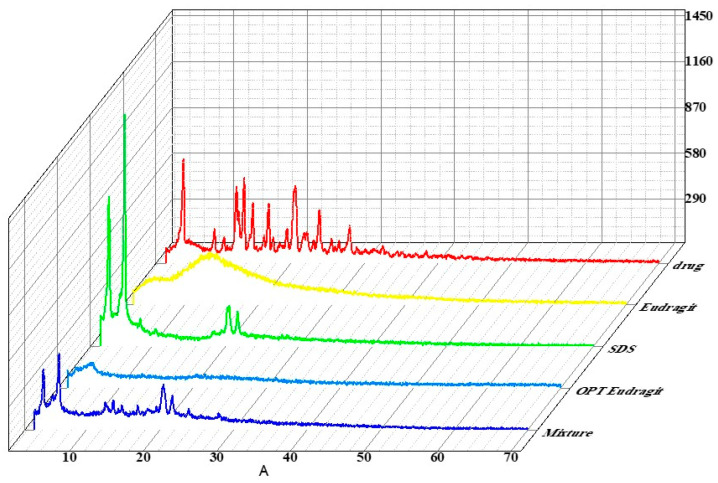
XRD spectra of drug-loaded Eudragit nanoparticles.

The XRD pattern of Eudragit exhibits no intense peaks, which confirmed its amorphous nature. SDS showed its characteristic peaks at 3° and 5° at 2θ, showing its crystalline nature. The XRD pattern of tamoxifen citrate-loaded Eudragit nanoparticles displayed no characteristic peaks of the drug, which confirmed that drug is present in amorphous form in the nanoparticles and might be dispersed in the polymeric matrix [46,47,48]. The XRD spectra of pure drug, Eudragit, SDS, physical mixture, and optimized formulation are shown in Figure 11.

### 3.20. Thermo-Gravimetric Analysis (TGA)

**Figure 12:**
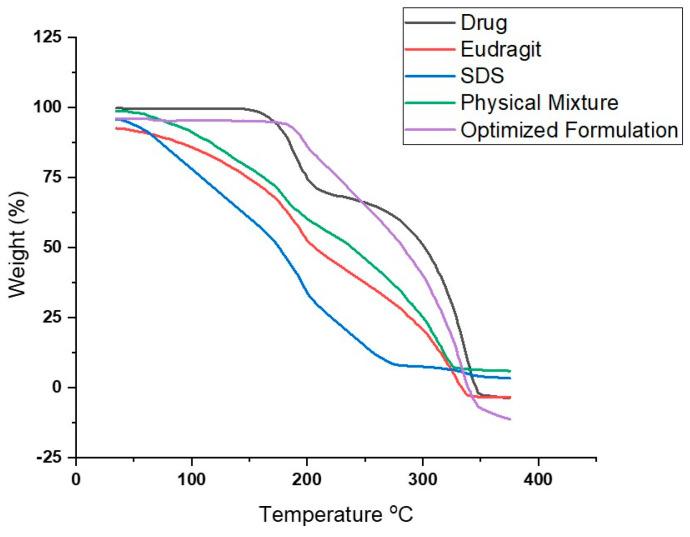
TGA Spectra; a. Drug, b. Eudragit, c. SDS, d. physical mixture, e. optimized Eudragit formulation.

The TGA curve of Eudragit nanoparticles also confirmed the thermal stability of the nanoformulation. The TGA curve of Eudragit starts to degrade at 90 °C and shows thermal stability at 320 °C. The thermal decomposition of Eudragit appears with a major peak at 370 °C, with a shoulder at 330 °C. The thermal decomposition of the surfactant (sodium dodecyl sulphate, SDS) starts at 200 °C, with a major peak of thermal decomposition at 230 °C. The physical mixture of drug, Eudragit, and surfactant starts to degrade at 190 °C, with a large thermal decomposition peak at 300 °C and a sharp shoulder at 200 °C. The thermal stability of drug-loaded Eudragit nanoparticles was confirmed via an analysis of the TGA curve. The thermal decomposition of optimized Eudragit nanoformulation starts at 200 °C with a large clean thermal decomposition peak at 235 °C up to 250 °C. The results confirmed that the thermal stability of tamoxifen encapsulated in the polymeric nanoparticles was not affected, as the thermal decomposition temperature of tamoxifen was not decreased; rather, the degradation temperature was enhanced slightly, though Eudragit is a low-melting-point polymer. The results indicated that the optimized formulation is thermally stable. The TGA spectra are shown in Figure 12.

### 3.21. In Vitro Drug Release Evaluation of Drug Loaded Eudragit Nanoparticles

The in vitro drug release profile of drug-loaded Eudragit nanoparticles was performed on a Franz cell using phosphate buffer (pH 7.4) as a dissolution media.

**Figure 13:**
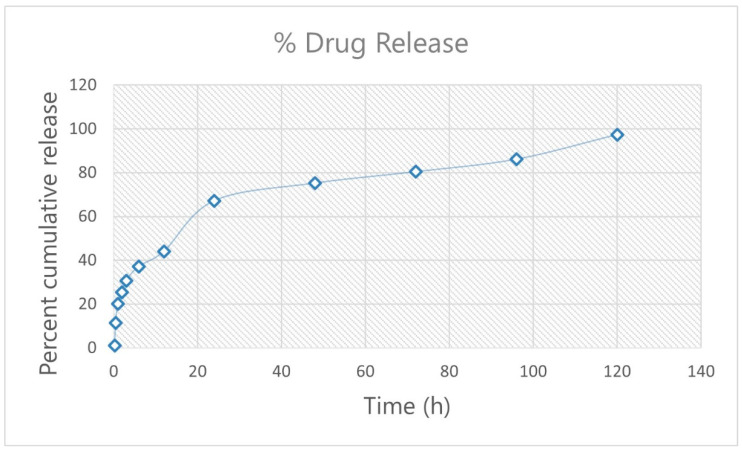
In vitro drug release profile of drug-loaded Eudragit nanoparticles.

The in vitro drug release profile of drug-loaded Eudragit nanoparticles is shown in Figure 13. The results recommended that the encapsulation of drug in Eudragit hinders the release of the drug, resulting in a confirmed biphasic drug release pattern, i.e., initial fast release of drug from the matrix (burst effect) followed by a linear dissolution profile. The results were confirmed by applying the data to different mathematical models; the results were in good agreement with the previous reports from the literature [49,50]. For up to 6 h, the fast release (burst effect) was seen, followed by a linear dissolution drug release profile until 120 hrs. The fast release is ascribed to the drug present on the surface of the nanoparticles.

### 3.22. In vitro Release Kinetics

Various pharmacokinetic models such as zero-order, first-order, Higuchi, Hixson, and the Crowell and Korsmeyer model were fitted to the data to study the kinetics and predict the possible drug release mechanism from the drug-loaded Eudragit nanoparticles. The results are shown in Table 12.

The data best fit to the Higuchi model, as is evident from the high R^2^ value of 0.948. Acknowledging that the drug release followed a diffusion process, the n value of 0.335 confirms the Fickian diffusion process of the drug-loaded Eudragit nanoparticles, as acknowledged by the Korsmeyer and Peppas model. The results of the dissolution models were in good agreement with previous literature [51,52,53,54]. The release of the drug was gradually decreased at the later stage of dissolution due to the low permeability of the Eudragit RS 100 [50]. The dissolution medium penetrates the nanoparticles, interacting with the dissolving Tam that then diffuses out through the pores via Fickian diffusion.

### 3.23. Stabilities Studies

To assess the storage effect on the PS, PDI, and EE of the nanoformulations, stability studies were carried out on the optimized drug-loaded polymeric (Eudragit) nanoformulations at two different temperatures, i.e., at refrigerated temperature (4 °C) and at room temperature (25 °C). The nanoformulation was stored for two months to examine the stability. At 4 °C, the observed changes in particle size, PDI and %EE were statistically insignificant compared with room temperature, at which the changes were significant. The results reveals that low temperature promotes less particle aggregation due to reduced kinetic energy, thus preventing particulate collision compared with high temperature, which promotes the aggregation of particles [55].

The results suggested that for physio-chemical stability, the nanoformulations should be stored at a refrigerated temperature of 4 °C. The results are shown in Table 13.

## 4. Conclusions

To demonstrate the application of statistical experimental designs in parameter optimization, an experimental example was chosen. Some factors were not examined further because they were thought to be invariant. For instance, the choice of stabilizer in a medicinal product would be seen as crucial to product quality, health and safety, and environmental considerations, and would need to be explicated and controlled. Tamoxifen-loaded Eudragit nanoparticles with a size of up to 200 nm and 90% EE for breast cancer were prepared and used as a model setting in this investigation. Based on our findings, it is recommended that incorporating the Eudragit nanoparticles in the oral dosage form will result in higher cell uptake and retention as it bears a negative charge, thus avoiding protein corona formation. They were created effectively by employing several Quality by Design principles. It was feasible to learn in general how each parameter affects each outcome, how substantial this influence is, and its direction using the screening strategy. With this information, it was possible to control the results while staying within the boundaries of the plan. Even if the screening design does not cover a certain desired outcome, it is still possible to determine which parameter must be altered further. As a result, a screening design is an excellent pretesting technique. A pharmaceutical product ought to have well-defined quality characteristics. A well-defined and regulated production process is essential as a gauge of Quality by Design, and vital process characteristics should be understood. The method presented here is a practical technique which allows us to accomplish this objective with minimal effort.

## Figures and Tables

**Table 1:** 2^3^ Factor design with the values of respective input factor.

Independent Factors	Levels		
	−1	0	1
Amount of the drug	1 mg	2 mg	3 mg
Amount of the polymer (Eudragit)	2.5 mg	5 mg	7.5 mg
Amount of SDS	0.1%	0.2%	0.3%

**Table 2:** Actual factor levels for the 2^3^ Factorial design with three center-points for Eudragit NPs.

Standard Run Order	Randomized Run Order	Conc. of Drug (mg)	Conc. of Eudragit (mg)	Conc. of Stabilizer (%) SDS
1	9	1	2.5	0.1
2	3	3	2.5	0.1
3	8	1	7.5	0.1
4	6	3	7.5	0.1
5	5	1	2.5	0.3
6	7	3	2.5	0.3
7	10	1	7.5	0.3
8	11	3	7.5	0.3
9	1	1	5	0.2
10	4	3	5	0.2
11	2	1	5	0.2

**Table 3:** Run parameter and responses of 2^3^ full factorial design.

Std	Run	Factor A Drug (mg)	Factor B Polymer (mg)	Factor C Stabilizer (%)	Responses	
					Particle Size (nm)	%EE
1	**9**	−1.00	−1.00	−1.00	187	94
2	**3**	1.00	−1.00	−1.00	142	79
3	**8**	−1.00	1.00	−1.00	149	95
4	**6**	1.00	1.00	−1.00	132	78
5	**5**	−1.00	−1.00	1.00	177	63
6	**7**	1.00	−1.00	1.00	142	72
7	**10**	−1.00	1.00	1.00	151	69
8	**11**	1.00	1.00	1.00	148	79
9	**1**	0.00	0.00	0.00	152	75
10	**4**	0.00	0.00	0.00	149	75
11	**2**	0.00	0.00	0.00	149	73

**Table 4:** Model terms included in equation based on their %contribution (e; excluded term, m; included term) for particle size.

	Term	Stdized Effects	Sum of Squares	%Contribution
**m**	A-drug	−25	1250	50.1861
**m**	B-polymer	−17	578	23.2061
**e**	C-surfactant	2	8	0.321191
**m**	AB	15	450	18.067
**e**	AC	6	72	2.89072
**e**	BC	7	98	3.93459
**e**	ABC	1	2	0.0802978
**e**	Curvature	−3.65563	26.7273	1.07307
**e**	Lack of Fit		0	0
**e**	Pure Error		6	0.240893

**Table 5:** ANOVA table of PS for drug-loaded Eudragit nanoparticles.

Source	Sum of Squares	df	Mean Square	F-Value	*p*-Value	
Model	2278.00	3	759.33	24.99	0.0004	significant
A-Drug	1250.00	1	1250.00	41.13	0.0004	
B-Polymer	578.00	1	578.00	19.02	0.0033	
AB	450.00	1	450.00	14.81	0.0063	
Residual	212.73	7	30.39			
Lack of Fit	206.73	5	41.35	13.78	0.0690	not significant
Pure Error	6.00	2	3.00			
Cor Total	2490.73	10				

**Table 6:** Descriptive statistics of PS for drug-loaded Eudragit nanoparticles.

Std. Dev.	5.51	R^2^	0.9146
Mean	152.55	Adjusted R^2^	0.8780
C.V. %	3.61	Predicted R^2^	0.7240
		Adeq Precision	12.6344

**Table 7:** Model terms included in equation based on their %contribution (e; excluded term, m; included term).

	Term	Stdized Effects	Sum of Squares	%Contribution
m	A-drug	−3.25	21.125	2.27462
e	B-polymer	−3.25	21.125	2.27462
m	C-surfactant	−15.75	496.125	53.4199
e	AB	0.25	0.125	0.0134593
m	AC	−12.75	325.125	35.0076
e	BC	3.25	21.125	2.27462
e	ABC	−0.75	1.125	0.121134
e	Curvature	−4.4825	40.1856	4.32695
e	Lack of Fit		0	0
e	Pure Error		2.66667	0.287131

**Table 8:** ANOVA of %EE for drug-loaded Eudragit nanoparticles.

Source	Sum of Squares	df	Mean Square	F-Value	*p*-Value	
**Model**	842.38	3	280.79	22.76	0.0005	significant
A-Drug	21.13	1	21.13	1.71	0.2320	
C-SDS	496.13	1	496.13	40.22	0.0004	significant
AC	325.12	1	325.12	26.36	0.0013	significant
**Residual**	86.35	7	12.34			
Lack of Fit	83.69	5	16.74	12.55	0.0754	not significant
Pure Error	2.67	2	1.33			
**Cor Total**	928.73	10				

**Table 9:** Descriptive statistics of %EE of drug-loaded Eudragit nanoparticles.

Std. Dev.	3.51	R^2^	0.9070
Mean	77.45	Adjusted R^2^	0.8672
C.V.%	4.53	Predicted R^2^	0.7529
		Adeq Precision	13.4562

**Table 10:** Optimized formulation factors level and experimental values of Eudragit nanoparticles.

Formulation Variables	Coded Value	Actual Values
Factor A (Drug)	−1.00	1 mg
Factor B (Polymer)	−1.00	2.5 mg
Factor C (Surfactant)	−1.00	0.1%
**Predicted Particle size**		**181 nm**
**Experimental Particle size**		**178 nm**
**Predicted %EE**		**93%**
**Experimental %EE**		**90%**
**Drug loading**		**43%**

**Table 11:** Confirmation on (95% confidence). Two-sided confidence = 95%.

Solution 1 of 87 Response	Predicted Mean	Predicted Median	Std Dev	n	SE Pred	95% PI low	Data Mean	95% PI High
PS	181.045	181.045	5.51268	1	6.67446	165.263	178	196.828
EE	93.3295	93.3295	3.51227	1	4.25247	83.274	90	103.385

**Table 12:** Results for model fitting to the fraction released data for drug-loaded Eudragit nanoparticles.

S. No	Model	R^2^	k	n
1	Zero order	0.812	0.684	--
2	First order	0.770	0.228	--
3	Hixson and Crowell	0.934	0.022	--
4	Higuchi	0.948	13.16	--
5	Korsmeyer and Peppas	0.959	--	0.335

R^2^ is the coefficient of determination. k is the proportionality constant. n is the exponent of release in function of time.

**Table 13:** Stability studies of optimized drug-loaded Eudragit. NPs stored at 4 °C and 25 °C.

Time	NPs	Stored at 4 °C				Stored at 25 °C			
		PS	PDI	Zeta	%EE	PS	PDI	Zeta	%EE
Day 1	**Drug-loaded** **Eudragit** **NPs**	178.7 ± 0.04	0.01 ± 0.04	−48.7 ± 0.09	90.3 ± 0.12	178.8 ± 0.11	0.13 ± 0.01	−48.6 ± 0.15	90.2 ± 0.12
Week 1		178.7 ± 0.16	0.01 ± 0.08	−48.5 ± 0.016	90.2 ± 0.16	185.4 ± 0.2	0.17 ± 0.02	−44.3 ± 0.20	89.7 ± 0.14
Week 2		177.7 ± 0.12	0.03 ± 0.03	−47.4 ± 0.20	89.6 ± 0.16	192.8 ± 0.58	0.23 ± 0.02	−41.5 ± 0.15	88.7 ± 0.24
Week 3		177.5 ± 0.12	0.13 ± 0.08	−46.6 ± 0.50	88.9 ± 0.16	202.6 ± 1.15	0.37 ± 0.01	−39.7 ± 0.23	87.7 ± 0.64
Week 4		177.7 ± 0.65	0.13 ± 0.04	−46.9 ± 0.28	88.5 ± 0.24	209.6 ± 0.41	0.40 ± 0.01	−35.5 ± 0.25	85.6 ± 0.36

## Data Availability

Data will be available upon request.
